# Factors associated with chronic pain clinical decision support use in primary care

**DOI:** 10.1371/journal.pdig.0001032

**Published:** 2026-07-16

**Authors:** Emma McCord, Nate C. Apathy, Justin Blackburn, Ann M. Holmes, Lindsey Sanner, Christopher A. Harle, Olena Mazurenko

**Affiliations:** 1 Department of Health Policy and Management, Fay W Boozman College of Public Health, University of Arkansas for Medical Sciences, Little Rock, Arkansas, United States of America; 2 Department of Health Policy and Management, Richard M. Fairbanks School of Public Health, Indiana University, Indianapolis, Indiana, United States of America; 3 Regenstrief Institute, Indianapolis, Indiana, United States of America; 4 Department of Health Policy and Management, School of Public Health, The University of Maryland, College Park, Maryland, United States of America; Yonsei University College of Medicine, KOREA, REPUBLIC OF

## Abstract

Clinical Decision Support (CDS), despite its potential to enhance healthcare quality, faces persistently low use. Prior research, often relying on self-reported data, has largely overlooked how clinician characteristics, such as gender and years in practice, moderate CDS use. We examined the moderating role of primary care clinician (PCC) characteristics on the relationship between patient-encounter characteristics and CDS use. We analyzed electronic health record data from a pragmatic randomized controlled trial (October 2019-May 2022) involving 69 PCCs with access to a CDS for managing chronic non-cancer pain (OneSheet). Our primary outcome was OneSheet use at a patient-encounter, defined as access within three days before or after an encounter. Independent variables included encounters for new patients, patients with chronic pain diagnoses, patients prescribed long-term opioid therapy (LTOT), and previous PCC OneSheet use. Generalized linear models with interaction terms tested PCC gender and years in practice as moderators. PCCs used OneSheet in 959 of 145,511 encounters (0.7%). Use was less likely for encounters with new patients (-0.42 percentage points (pp); 95% confidence interval (CI) [-0.65, -0.19]) but more likely for encounters with chronic pain diagnoses (3.48 pp; 95% CI [2.62, 4.33]) and patients prescribed LTOT (4.70 pp; 95% CI [3.57, 5.84]). Prior OneSheet use did not predict future use (0.30 pp; 95% CI [-0.00, 0.06]). Relationships were strongest among female PCCs with over 16 years of practice. Optimizing CDS use requires prioritizing clinical relevance and considering the diverse characteristics of clinicians.

## Author summary

Clinical decision support (CDS) are digital tools designed to help clinicians make evidence-based decisions during patient care. While these tools hold great promise for improving healthcare quality, they are rarely used in everyday practice. Much of what we know about CDS use comes from clinician surveys, which may not capture real-world patterns of use or how clinicians’ own backgrounds influence adoption. In this study, we examined how primary care clinicians’ (PCCs) characteristics, such as gender and years in practice, shape their use of a CDS (OneSheet) for managing chronic pain. Using electronic health record data from more than 145,000 patient encounters across a randomized trial, we found that PCCs used the tool in less than 1% of encounters. Usage was more common when patients had chronic pain diagnoses or long-term opioid prescriptions, and less common with new patients. Importantly, these patterns were most pronounced among female clinicians with longer experience in practice. Our findings highlight that simply providing CDS tools is not enough. Efforts to increase adoption must ensure the CDS are clinically relevant and consider the diversity of clinicians who use them.

## 1. Introduction

Clinical decision support (CDS) are digital resources that use clinical knowledge and patient information to support clinical decision-making, which can enhance healthcare quality and safety through evidence-based practices and streamline workflows [1–17]. Despite its potential, CDS is used in less than half of appropriate cases [18]. Several barriers and facilitators to CDS use have been identified using self-reported data from end-users, which are susceptible to recall and social desirability bias and may not accurately reflect actual use patterns [19–26]. Furthermore, these studies did not account for end-user characteristics, such as gender and years in practice that may be associated with or moderate CDS use. Ignoring end-user characteristics oversimplifies the identification of factors affecting CDS use, leading to generic implementation strategies that fail to address user diversity.

Understanding the moderating effects of end-user characteristics is particularly relevant for CDS aimed at assisting primary care clinicians (PCCs) caring for patients with complex needs under time and information constraints, such as chronic non-cancer pain (CNCP) [27–30]. For instance, when prescribing opioids for patients with CNCP, PCCs must review data from the prescription drug monitoring program, a process that CDS can support by streamlining data access and documentation [31–33]. Prior qualitative work has demonstrated that certain patient-encounter characteristics (e.g., new patient, or a patient with complex medical histories, etc.), influenced PCCs’ decision to use a CDS for chronic pain management (i.e., OneSheet) [20,24]. Notably, qualitative work is limited in the ability to investigate potential effects of PCC characteristics, such as gender and years in practice, in moderating the relationship between patient-encounter characteristics and CDS use. However, the way these encounter characteristics translate into actual CDS use likely varies across clinicians because PCC characteristics, such as experience and gender, shape perceptions of usefulness, workflow fit, and effort. Understanding the moderating effects of PCC characteristics is crucial for developing approaches which accommodate user preferences and enhance CDS use for chronic pain management.

We used the Unified Theory of Acceptance and Use of Technology (UTAUT) framework and real-world electronic health record (EHR) data to examine how PCC characteristics moderate the relationship between patient-encounter characteristics and CDS use in primary care [34,35]. The UTAUT framework explicitly conceptualizes how individual user characteristics influence the relationship between contextual factors and technology use, aligning directly with our moderation-focused research questions [34,35]. To answer our research questions, we used EHR data from a randomized controlled trial (RCT), in which we assessed the effect of OneSheet on guideline-recommended chronic pain management in primary care at two health systems from October 2020 to May 2022 [36]. Our findings can inform tailored training, workflow integration, CDS design, helping health systems optimize adoption, clinicians’ use of CDS more strategically, and developers to build tools that are both user-friendly and clinically relevant.

### 1.1. Conceptual framework

Our study draws on UTAUT, a framework developed and commonly used by information systems researchers to explain why individuals adopt and use new technologies [34]. UTAUT synthesizes eight prominent models of behavior or technology acceptance from the psychology and information systems literatures into a single, comprehensive framework [34]. The core UTAUT model specifies four primary constructs that shape technology use: performance expectancy (perceived usefulness), effort expectancy (ease of use), social influence (extent to which individuals believe those in their social environment expect them to use the technology), and facilitating conditions (existence of organizational and technical infrastructure to facilitate the use of the technology) [34,35]. In this analysis, we focus on performance and effort expectancy because these constructs can be reasonably examined using EHR audit log data. Although social influence and facilitating conditions are important in shaping technology use, we were unable to directly measure these constructs using the available EHR data and therefore did not include them in our analysis.

Thus, we adapt the core UTAUT framework constructs to model how PCC characteristics moderate the relationship between patient-encounter characteristics and CDS use [34,35,37–42]. UTAUT posits that user characteristics influence perceived usefulness and ease of use, which ultimately drives technology use [34,35,43]. This moderating role is often overlooked but salient in the CDS context as its value may not be uniformly perceived [37–44]. For example, inexperienced users may avoid a more complex, workflow-specific CDS as they focus on establishing more generalized workflows and familiarizing themselves with a CDS that applies to most of their encounters (e.g., drug-drug interaction alerts). Conversely, more experienced users, already familiar with core CDS functions, may be better positioned to adopt a more specialized CDS that aligns with specific clinical workflows [43,45]. Moreover, gender differences in technology use have been documented [45]. Although data specific to CDS usage by gender are limited, patterns observed in EHR use offer meaningful insights. Female clinicians spend more time on documentation and after-hours EHR tasks, produce longer, more detailed notes, and handle a higher volume of messages and complex tasking; thus, they are likely to use CDS differently than their male counterparts [46–52]. Furthermore, clinician gender influences not only how clinicians use CDS, but also how they perceive their efficiency, workload, and overall utility.

We supplement the UTAUT model by incorporating patient-encounter characteristics as key factors that influence how PCCs use CDS. Prior qualitative work identified specific patient-encounter characteristics that shaped PCCs decision to use a CDS for chronic pain management (i.e., OneSheet). OneSheet streamlines guideline-relevant information (e.g., pain diagnoses, pain and function goals, etc.) into a single EHR view and provides shortcuts to recommended clinical actions (e.g., ordering urine drug screenings) [53]. OneSheet reduces the need to search across multiple EHR sections, helping PCCs quickly access comprehensive, relevant information in one place, ultimately improving efficiency and supporting better-informed decision-making (i.e., higher performance expectancy). However, during shorter or non-chronic pain-related encounters, OneSheet may be irrelevant (i.e., lower performance expectancy).

**Fig 1:**
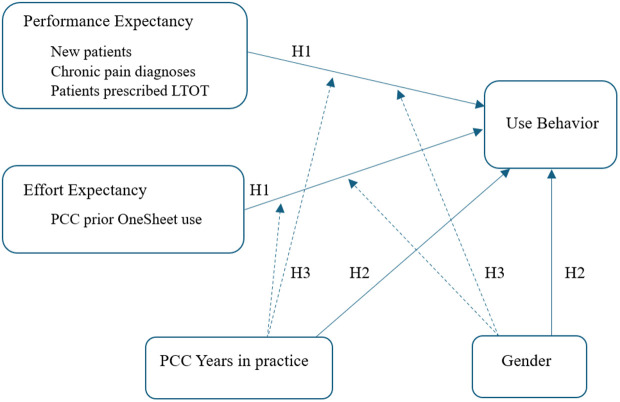
Proposed research model and hypotheses.

Our conceptual framework (see Fig 1) structures our examination of “by whom and for whom” CDS are used by accounting for both the nature of patient-encounters and PCC characteristics. Specifically, we propose that the influence of patient-encounter characteristics on CDS use depends on PCC gender and years in practice (i.e., experience). This framework offers a more comprehensive understanding of CDS use, showing how PCC characteristics and patient-encounter characteristics interact to shape real-world use. It can also help inform strategies for tailoring CDS design and implementation to meet the needs of different types of users.

Based on this framework, we propose the following hypotheses:

*Hypothesis 1: CDS use is associated with patient-encounter characteristics related to performance (i.e., encounters with new patients, an attached chronic pain diagnosis, or patients prescribed LTOT) and effort expectancy (e.g., PCCs previous CDS use) measures.*

*Hypothesis 2: CDS use is associated with PCC experience and gender.*

*Hypothesis 3: The association between patient-encounter characteristics and CDS use is moderated by PCC experience and gender.*

## 2. Materials and methods

We analyzed secondary data from a pragmatic RCT conducted from October 2020 through May 2022. The purpose of this RCT was to assess the effect of OneSheet on guideline-recommended chronic pain management in primary care. OneSheet was developed through user-centered design research, which included PCC interviews, patient observations, and prototype testing. Created during heightened scrutiny of opioid prescribing for CNCP and the release of national guidelines, OneSheet addresses PCCs’ decision-making challenges driven by knowledge gaps and limited training in chronic pain management. The RCT included 137 PCCs across 25 clinics and two study sites; 69 were randomized to treatment and had access to OneSheet in their Epic EHRs. Additional details of our recruitment strategy and trial design are reported elsewhere [36,53–55].

### 2.1. Ethics statement

The Indiana University Institutional Review Board approved all study procedures before the study’s commencement, including informed consent for participating PCCs and a waiver of consent for patients. Patient data was already routinely collected in the Epic EHR system, presenting no additional privacy risks for patients. Furthermore, the high volume of patients with chronic pain seen daily made obtaining individual consent and HIPAA authorization impractical. The study is registered with ClinicalTrials.gov (NCT04295135).

### 2.2. Setting and participants

**Fig 2:**
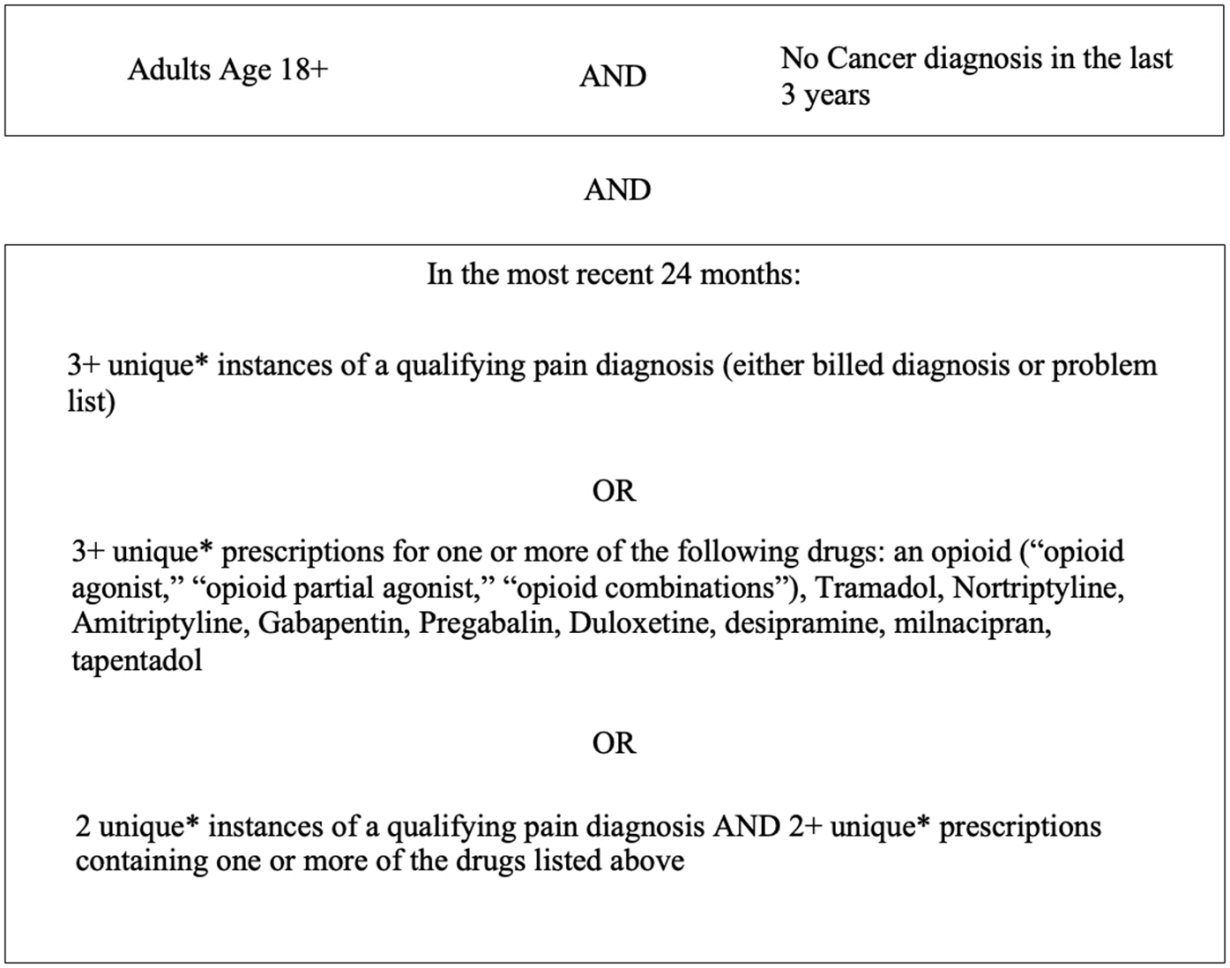
Approach for identifying patients with chronic pain.

We used encounter-level EHR data for PCCs with OneSheet access for encounters with patients diagnosed with chronic pain after OneSheet was implemented, identified using a pre-defined algorithm (see Fig 2). PCC demographic information (i.e., sex, race, ethnicity, clinical training, and years in practice) was recorded at enrollment in the RCT.

### 2.3. Dependent variable

**Fig 3:**
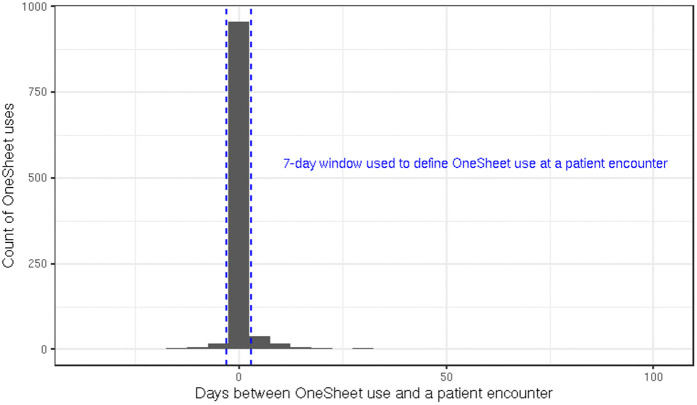
Distribution of OneSheet uses for patients in our sample.

To measure OneSheet use, we analyzed EHR audit log data, which contained the unique PCC ID and corresponding access date. We created a variable for OneSheet use at a patient-encounter by matching PCCs’ unique OneSheet access to individual patient-encounters. We identified the closest patient-encounter date to a given OneSheet use date by a PCC to attribute it to only one encounter. OneSheet use attributed to a patient-encounter was defined as use by PCCs within a 7-day patient-encounter window: 3 days before, on the day of, or up to 3 days after a patient-encounter (see Fig 3). This window was chosen based on the distribution of patient-encounters closest to the access date, which corresponds to qualitative evidence from clinicians regarding their workflows for preparing for and responding to patient-encounter needs [26]. When multiple encounters occurred within this 7-day patient-encounter window, OneSheet access was assigned to the encounter closest in time to the access date. This approach ensured that each OneSheet access was attributed to a single encounter, though it may result in conservative estimates of use for patients with frequent encounters. We considered OneSheet use outside this 7-day patient-encounter window to be non-encounter-related.

### 2.4. Independent variables

The key independent variables were patient-encounter characteristics associated with performance and effort expectancy, and PCC characteristics. We operationalized performance and effort expectancy using the following patient-encounter characteristics: 1) a patient was new to a PCC (yes/no); 2) a chronic pain diagnosis was attached to the patient-encounter (yes/no); 3) a patient was prescribed long-term opioid therapy (LTOT) (yes/no); and 4) PCCs previous OneSheet use. Encounter data were grouped by patient and PCC identifiers and sorted by encounter date to identify the first observed patient-PCC match during our study period. This encounter was coded as “yes” to indicate the patient was new to a PCC. We extracted pain diagnoses from the EHR and identified encounters with a chronic pain diagnosis attached (i.e., primary or secondary) where OneSheet was used. This served as a proxy for the patient’s chief complaint, which was chronic pain. We extracted reason for encounter data from the scheduling module but found these data to be unreliable (i.e., missing or nonspecific), leading us to rely on the diagnosis data. An encounter resulting in a patient prescribed LTOT was defined as a prescription of at least a 28-day supply of an opioid analgesic at the encounter [22]. Previous PCC OneSheet use was measured as the cumulative number of previous encounters in which the PCC had used OneSheet for any patient. PCC characteristics included gender (i.e., male or female) and years in practice (i.e., time since completing medical training), both self-reported in a baseline enrollment survey [54].

### 2.5. Analysis

We used descriptive statistics, t-tests, and chi-square tests to compare patient-encounter characteristics stratified by OneSheet use at a patient-encounter. Four progressive generalized linear models examined how PCC characteristics modify CDS use, isolating individual and joint effects of PCC characteristics on CDS use. Models sequentially added: 1) patient-encounter characteristics; 2) PCC years in practice and interactions; 3) PCC gender and interactions (removing years in practice); and 4) all variables. Statistical tests were two-tailed (p < 0.05). Data analyses were performed using the *tidyverse, stats, lmtest, mfx,* and *margins* packages in R (version 4.4.1) [56–60]. To better understand the clinical significance of our findings, we converted marginal effect estimates to an equivalent numbers needed to treat (i.e., the number of eligible patient-encounters associated with one additional OneSheet use).

Due to the multi-level nature of our data, wherein encounters are nested within patients who are nested within PCCs, we estimated an intraclass correlation coefficient (ICC) with PCC random effects and patient random effects. S1 Table presents the ICC estimates, indicating variance in OneSheet use attributable to PCCs or patients. ICC values (<0.2) suggested minimal variability attributable to PCC or patient characteristics. Since no significant clustering was found, we chose not to present a multi-level model with random effects as our primary analysis. Methodological literature suggests that when ICCs are low, single-level models produce unbiased estimates, particularly when the primary objective is estimating associations rather than partitioning variance across levels [61,62].

### 2.6. Sensitivity analysis

Three separate models assessed the potential impact of two PCC outliers for years in practice (30 and 42 years). Models excluded one outlier PCC, the other PCC, or both PCCs, to examine the individual and combined effects of outlier PCCs.

## 3. Results

PCCs used OneSheet for 959 encounters out of a total of 145,511 encounters with patients with chronic pain included in our study (0.7%) (see Table 1). OneSheet was rarely used in encounters with new patients (0.2%, p < 0.001) but was more frequently used for encounters involving patients with an attached chronic pain diagnosis (2.4%, p < 0.001) or patients prescribed LTOT (3.8%, p < 0.001). On average, PCCs used OneSheet fewer times prior to encounters where it was used compared to those where it was not (mean 4.18 vs. 4.38, p = 0.019).

**Table 1:** Patient-encounter characteristics stratified by OneSheet use.

Characteristic	Total encounters (n = 145,511)	OneSheet used at encounter (n = 959)	OneSheet not used at encounter (n = 144,552)	P-value
Patient is new to PCC (Reference: No), n(%)	8,388	18 (0.2)	8,370 (99.8)	<0.001
Chronic pain diagnosis attached to encounter (Reference: No), n(%)	28,932	700 (2.4)	28,232 (97.6)	<0.001
Patient is prescribed LTOT (Reference: No), n(%)	15,480	582 (3.76)	14,898 (96.24)	<0.001
PCC previous OneSheet uses (SD)	4.38 (2.74)	4.18 (2.86)	4.38 (2.74)	0.019

Note: PCC – primary care clinician; LTOT – Long-term opioid therapy; SD – Standard deviation.

### 3.1. Primary analysis

#### 3.1.1. Model fit.

Model fit was evaluated using log-likelihood (LL) and likelihood-ratio (LR) tests. Model 4 (LL = -4,274.80) demonstrated a better fit than Model 1 (LL = -4,682.10), with the LR test (814.60, p < 0.001) indicating this improvement is statistically significant. Therefore, we focus on results from Model 4, which demonstrated the best overall fit.

#### 3.1.2. Patient-encounter characteristics associated with performance and effort expectancy measures.

We found partial support for hypothesis 1. PCCs were less likely to use OneSheet for encounters with new patients (-0.42 percentage point (pp), 95% Confidence Interval (CI) [-0.65, -0.19]). PCCs were more likely to use OneSheet for encounters with an attached chronic pain diagnosis (3.48 pp, 95% CI [2.62, 4.33]), and patients prescribed LTOT (4.70 pp, 95% CI [3.57, 5.84]) (see Table 2). PCCs previous OneSheet use was not associated with OneSheet use at a patient-encounter (0.03 pp, 95% CI [–0.00, 0.06]).

**Table 2:** Generalized linear model results, performance expectancy, effort expectancy, and moderating factors associated with using OneSheet at a patient-encounter.

Variable	Model 1 ME (95% CI)	Model 2 ME (95% CI)	Model 3 ME (95% CI)	Model 4 ME (95% CI)
***Performance expectancy***				
Patient is new to PCC (Reference: No)	−0.00371*** (−0.00516, −0.00225)	−0.00496*** (−0.00667, −0.00324)	−0.00429*** (−0.00574, −0.00284)	−0.00419*** (−0.00645, −0.00193)
Pain diagnosis attached to encounter (Reference: No)	0.01418*** (0.01282, 0.01554)	0.03944*** (0.03080, 0.04808)	0.01905*** (0.01677, 0.02134)	0.03476*** (0.02621, 0.04331)
Patient is prescribed LTOT (Reference: No)	0.01991*** (0.01789, 0.02194)	0.05822*** (0.04619, 0.07025)	0.03055*** (0.02710, 0.03399)	0.04701*** (0.03565, 0.05837)
***Effort expectancy***				
PCC previous OneSheet uses	−0.00012 (−0.00027, 0.00003)	0.00013 (−0.00017, 0.00043)	−0.00004 (−0.00020, 0.00012)	0.00030 (−0.00004, 0.00064)
***PCC Characteristics***				
Years in practice		0.00052*** (0.00042, 0.00062)		0.00077*** (0.00063, 0.00091)
Male (Reference: female)			0.00218 (−0.00069,0.00506)	−0.00687*** (−0.00957, −0.00417)
***Testing for moderation***				
Patient is new to PCC* Years in practice		0.00018 (−0.0008, 0.00043)		0.00005 (−0.00030, 0.00040)
Pain diagnosis attached to encounter*Years in practice		−0.00043*** (−0.00053, −0.00034)		−0.00034*** (−0.00046, −0.00022)
Patient is prescribed LTOT*Years in practice		−0.00043*** (−0.00052, −0.00033)		−0.00031*** (−0.00043, −0.00019)
PCC previous OneSheet uses*Years in practice		−0.00001 (−0.00003,0.00000)		−0.00002* (−0.00004, −0.00000)
Patient is new to PCC* Male			0.00564 (−0.00564, 0.01694)	0.00429 (−0.00927, 0.01785)
Pain diagnosis attached to encounter*Male			−0.00640*** (−0.00759, −0.00521)	−0.00375*** (−0.00550, −0.00201)
Patient is prescribed LTOT* Male			−0.00520*** (−0.00642, −0.00399)	−0.00281** (−0.00475, −0.00087)
PCC previous OneSheet uses* Male			−0.00045* (−0.00085, −0.00004)	−0.00004 (−0.00052, 0.00043)
**Log likelihood**	−4,682.1	−4,563.7	−4,375.0	−4,274.8
**Likelihood Ratio Test**	2,179.6	236.8	614.2	814.6
**n**	145,511	145,511	145,511	145,511

Note: Because OneSheet use occurred in fewer than 1% of encounters, estimates should be interpreted as marginal effects on rare outcomes. While the large sample size supports model stability, small absolute effect sizes are expected in this context. Marginal effects derived from the mfx package in R. The mfx package provides the overall marginal effect for interaction terms. ME – marginal effects; CI – confidence interval; PCC – primary care clinician; LTOT – long-term opioid therapy; *p < 0.05, **p < 0.01, ***p < 0.001

#### 3.1.3. PCC characteristics.

Hypothesis 2 was fully supported. OneSheet use by PCCs was associated with having more years in practice, such that each year of practice increased the likelihood of OneSheet use by 0.08 percentage points (95% CI [0.06, 0.09]). Compared to females, male PCCs were less likely to use OneSheet during a patient-encounter (-0.69 pp, 95% CI [-0.96, -0.42]).

#### 3.1.4. Moderation analysis.

We found partial support for hypothesis 3. Moderation analyses identified several statistically significant interactions between PCC characteristics and patient-encounter characteristics. The likelihood of OneSheet use association varies depending on the PCCs years in practice (see Table 2) such that use is less likely for a patient-encounter with an attached chronic pain diagnosis (-0.03 pp, 95% CI [-0.05, -0.02]), for a patient prescribed LTOT (-0.03 pp, 95% CI [-0.04, -0.02]), or a PCCs previous OneSheet use (-0.002 pp, 95% CI [-0.004, -0.000]). The likelihood of OneSheet use association also varies by PCC gender with use less likely for an encounter with an attached chronic pain diagnosis (-0.38 pp, 95% CI [-0.55, -0.20]) or for a patient prescribed LTOT (-0.28 pp, 95% CI [-0.48, -0.09]) and OneSheet use.

**Fig 4:**
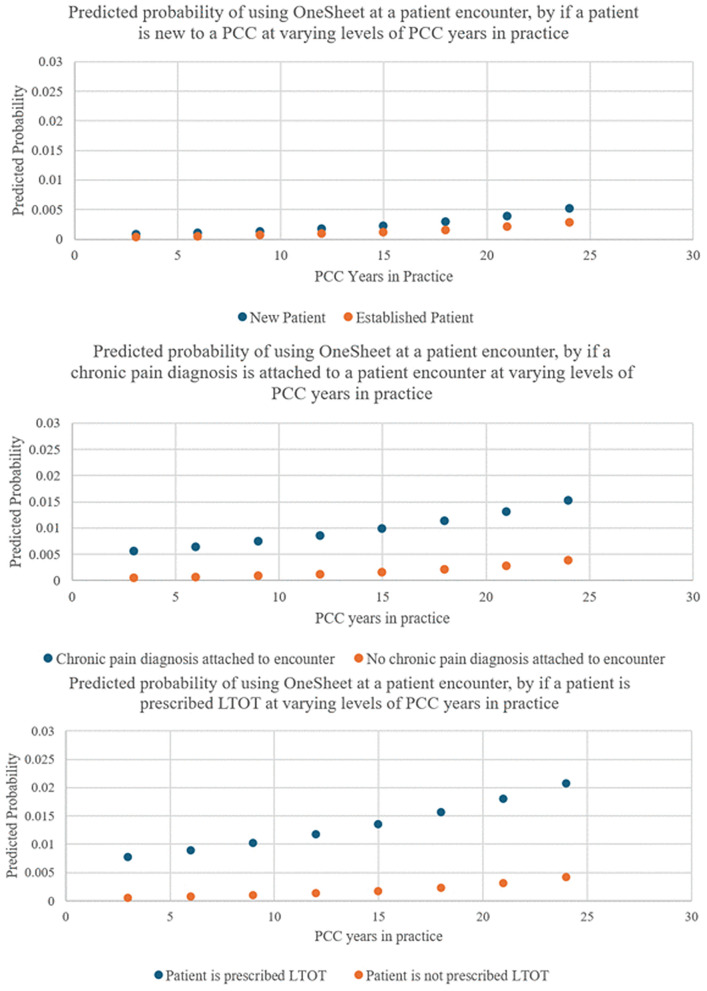
Predicted probability plots for interaction of performance expectancy measures and PCC years in practice.

**Fig 5:**
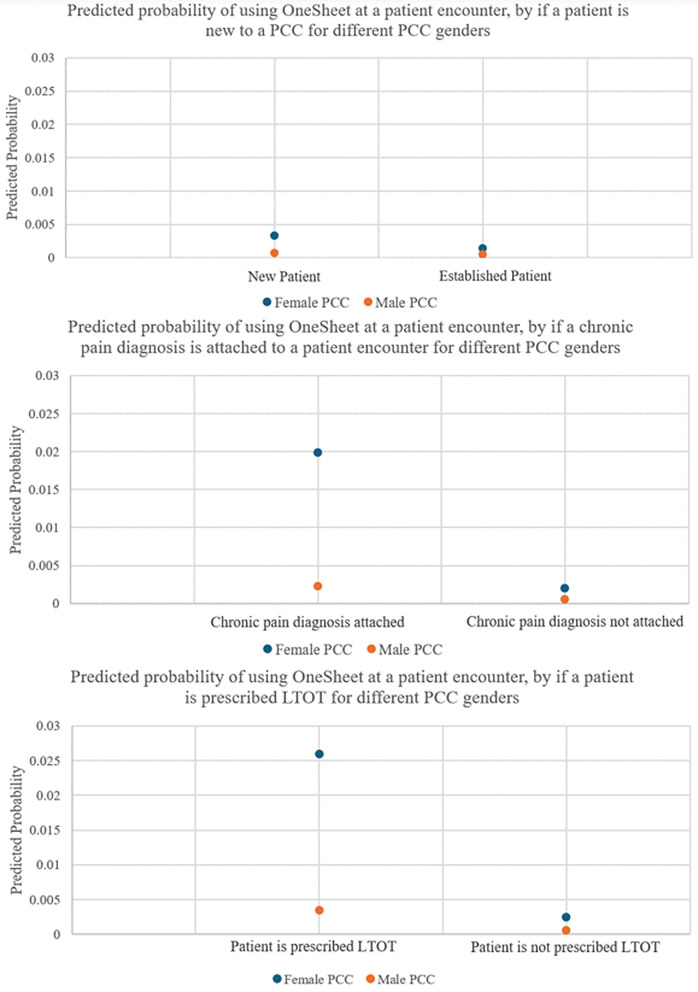
Predicted probability plots for interaction of performance expectancy measures and PCC gender.

We calculated predicted marginal effects and observed that PCC years in practice and gender affected the association between several patient-encounter characteristics and OneSheet use, which was statistically significant. More experienced PCCs were more likely to use OneSheet for encounters with an attached chronic pain diagnosis or patients prescribed LTOT (see Fig 4). For example, the likelihood of Onesheet use at an encounter for a patient prescribed LTOT increased by 2.2 percentage points for PCCs with 6 years in practice (95% CI [1.87, 2.59]) and by 2.6 percentage points for PCCs with 16 years in practice (95% CI [2.32, 2.85]). However, more experienced PCCs were less likely to use OneSheet for encounters with new patients. Female PCCs were more likely than males to use OneSheet for encounters with an attached chronic pain diagnosis (2.6 pp, 95% CI [2.29, 2.94] vs. 0.20 pp, 95% [0.11, 0.29]) or for patients prescribed LTOT (3.9 pp, 95% CI [3.42, 4.29] vs. 0.36 pp, 95% CI [0.23, 0.49]) (see Fig 5). PCC characteristics did not influence the relationship between PCCs previous OneSheet use and OneSheet use at a patient-encounter (see S2 Table for full results of moderation analysis).

#### 3.1.5. Clinical significance.

Encounters with new patients were associated with one fewer OneSheet use per 99 encounters. In contrast, encounters with an attached chronic pain diagnosis or a patient prescribed LTOT were each associated with one additional OneSheet use per 97 encounters. For PCC characteristics, each additional year in practice corresponded to one additional OneSheet use per 794 encounters, whereas encounters with male PCCs were associated with one fewer OneSheet use per 74 encounters.

### 3.2. Sensitivity analysis

S3 Table summarizes the results of the outlier sensitivity analysis. The first model (excluded one outlier PCC) closely mirrors the primary analysis, showing no substantial differences. However, the second and third models (excluded the other PCC and both outlier PCCs, respectively) differ from the primary analysis, particularly in testing for moderation. In these models, the interaction terms, *chronic pain diagnosis attached to encounter × years in practice* and *PCC previous OneSheet uses × years in practice*, are not statistically significant. In contrast, they were statistically significant in the primary analysis. This suggests that some moderation effects, particularly those involving years in practice, may be driven by a small number of highly experienced PCCs. While the direction of effects remained consistent, this indicates that conclusions regarding moderation by PCC experience should be interpreted cautiously.

## 4. Discussion

In this secondary analysis of EHR audit-log data from a pragmatic RCT, we found that CDS use for chronic pain management was rare but more likely during encounters with high clinical relevance, such as those involving patients with a chronic pain diagnosis or prescribed LTOT. Importantly, PCC characteristics, such as years in practice and gender, moderated these relationships, indicating that CDS use varies systematically by both user and context. Taken together, and in line with UTAUT, these results indicate that clinically relevant encounters heighten performance expectancy, while PCC characteristics shape how strongly that expectancy translates into actual CDS use, helping to explain both the persistently low uptake and the structured variation discussed below.

Specifically, we found that PCC characteristics, such as years in practice and gender, moderate the relationship between patient-encounter characteristics associated with performance expectancy (e.g., new patient, patient with a chronic pain diagnosis, and patient prescribed LTOT) and CDS use in statistically significant ways. Overall, PCCs with more experience were more likely to use OneSheet. However, less experienced PCCs tend to use it in situations where its use may be especially appropriate, such as patients with a chronic pain diagnosis or patients prescribed LTOT. This more targeted use may lead to greater value in specific contexts. Future research should examine why less experienced PCCs are more selective in their use. For example, are they more cautious, time-constrained, or discerning in identifying high-utility encounters?

Additionally, we found that more experienced PCCs were more likely to use OneSheet during encounters requiring synthesis of complex information. This finding fits with the demanding nature of chronic pain care, where experienced clinicians, having repeatedly navigated its intricacies, likely recognize OneSheet’s value in streamlining data [43,45]. We extend existing literature by showing that the perceived usefulness of a specialized CDS in cognitively demanding situations resonates more strongly with experienced users who can better integrate it into their established workflows [63,64]. This differentiated use is relevant given documented gender disparities in EHR workload and burnout [46–52]. If less experienced clinicians, potentially female or early career, adopt efficiency-enhancing CDS less, existing workload imbalances could worsen, leaving them without digital support that experienced colleagues use. Therefore, our findings highlight the need for CDS developers and health systems to promote equitable adoption across all experience levels and demographics, ensuring that technological advancements contribute to a more efficient and equitable clinical environment.

CDS tools are most likely to be used when their capabilities align with PCCs’ needs during specific patient-encounters. Although overall OneSheet use was low, involving only 1% of encounters with patients with chronic pain, usage increased during encounters with an attached chronic pain diagnosis or patients prescribed LTOT. This suggests PCCs turn to CDS when it offers meaningful support for accurate diagnoses and efficiency [37–42]. However, a critical barrier is that these tools rarely precisely reflect “highest alignment” encounters where information synthesis is paramount. Instead, a common aversion to false negatives (e.g., no CDS when it was relevant) leads to overly broad alert triggers and widespread “alert fatigue,” diminishing CDS utility and integration into workflows [65,66]. Thus, improving CDS use requires a user-centered design with context-specific alerting and targeted training to support clinician trust and workflow integration.

Furthermore, familiarity alone does not drive CDS use; contrary to expectations, previous OneSheet use did not increase future use, indicating initial use is insufficient for long-term use. This aligns with human factors research showing complex interfaces and information overload can reduce engagement [16,67]. While effort expectancy is important [37,38], our findings suggest that seamless integration into clinical workflows and ease of use may be more critical. If a CDS is too complicated or disrupts workflows, prior users may opt against it.

Our study has several strengths, including the use of real-world data from a multi-site pragmatic RCT and the application of UTAUT constructs informed by prior qualitative research to analyze actual CDS use. However, potential selection bias remains, as clinicians who did not use OneSheet may differ in unmeasured ways. Additionally, due to data availability, we were unable to assess all UTAUT constructs, such as social influence and facilitating conditions, which may also impact CDS use. As a result, our findings may not capture all factors influencing CDS use or fully generalize to settings with different organizational dynamics.

## 5. Conclusion

Despite CDS promise in chronic pain management, its use in primary care remains low. While overall use was limited, CDS use increased for encounters with attached chronic pain diagnoses or patients prescribed LTOT, suggesting its value in highly relevant contexts. Importantly, CDS adoption varies by PCC characteristics (years in practice, gender), underscoring the need for CDS and implementation strategies that cater to diverse users. By applying UTAUT, we show usability and workflow fit are more important than familiarity alone. Targeted training and clinician champions can further support adoption.

## Supporting information

S1 Table Intraclass correlation coefficients (ICC) estimates with PCC and patient random effects. (DOCX)

S2 Table Generalizes linear model results. (DOCX)

S3 Table Results of outlier sensitivity analysis. (DOCX)
