# Supplementary material for: Factors associated with chronic pain clinical decision support use in primary care
Source: PLOS Digit Health. 2026 Jul 16;5(7):e0001032. doi: 10.1371/journal.pdig.0001032 (PMC13374887; doi:10.1371/journal.pdig.0001032)
Supplement: S1 Table — (DOCX) [file pdig.0001032.s001.docx]

**S1 Table.** Intraclass correlation coefficient (ICC) estimates with PCC and patient random effects

| PCC Random effects | | | | |
| --- | --- | --- | --- | --- |
| ICC estimate | Null model | PCC model | Encounter model | Full model |
| Adjusted | 0.044 | 0.040 | 0.038 | 0.039 |
| Unadjusted | 0.044 | 0.040 | 0.037 | 0.038 |
| Patient Random effects | | | | |
| Adjusted | 0.145 | 0.144 | 0.137 | 0.132 |
| Unadjusted | 0.145 | 0.143 | 0.136 | 0.130 |

Note: Null model included no variables. The PCC model included PCC gender and years in practice. The encounter model included PCC gender, years in practice, if a patient was new to a PCC, if an encounter had a pain diagnosis attached, if an encounter was with a patient prescribed LTOT, and PCCs previous OneSheet uses. The full model included the variables from the encounter model as well as the interaction terms.
