# Supplementary material for: Factors associated with chronic pain clinical decision support use in primary care
Source: PLOS Digit Health. 2026 Jul 16;5(7):e0001032. doi: 10.1371/journal.pdig.0001032 (PMC13374887; doi:10.1371/journal.pdig.0001032)
Supplement: S2 Table — (DOCX) [file pdig.0001032.s002.docx]

**S2 Table**. Generalized linear model results

| Variable | Marginal effects (95% CI) |
| --- | --- |
| *Performance expectancy* |  |
| Patient is new to PCC (Reference: No) | -0.0034*** (-0.00512, -0.00170) |
| Chronic pain diagnosis attached to encounter (Reference: No) | 0.0144*** (0.01210, 0.01575) |
| Patient is prescribed LTOT (Reference: No) | 0.0238*** (0.02111, 0.02647) |
| *Effort expectancy* |  |
| PCC previous OneSheet uses | -0.0000 (-0.00002, 0.00004) |
| *PCC Characteristics* |  |
| Years in practice | 0.0003*** (0.00019, 0.00031) |
| Male (Reference: female) | -0.0118*** (-0.01306, -0.01047) |
| *Testing for moderation* |  |
| Patient is new to PCC* Years in practice |  |
| Yes*6 years in practice (Reference: No*6 years in practice) | -0.0030*** (-0.00445, -0.00150) |
| Yes*12 years in practice (Reference: No*12 years in practice) | -0.0034*** (-0.00486, -0.00203) |
| Yes*16 years in practice (Reference: No*16 years in practice) | -0.0039*** (0.00566, -0.00216) |
| Chronic pain diagnosis attached to encounter*Years in practice |  |
| Yes*6 years in practice (Reference: No*6 years in practice) | 0.0134*** (0.01161, 0.01520) |
| Yes*12 years in practice (Reference: No*12 years in practice) | 0.0153*** (0.01381, 0.01679) |
| Yes*16 years in practice (Reference: No*16 years in practice) | 0.0168*** (0.01511, 0.01851) |
| Patient is prescribed LTOT*Years in practice |  |
| Yes*6 years in practice (Reference: No*6 years in practice) | 0.0223*** (0.01874, 0.02585) |
| Yes*12 years in practice (Reference: No*12 years in practice) | 0.0242*** (0.02151, 0.02699) |
| Yes*16 years in practice (Reference: No*16 years in practice) | 0.0259*** (0.02322, 0.02845) |
| PCC previous OneSheet uses*Years in practice |  |
| Yes*6 years in practice (Reference: No*6 years in practice) | 0.0001 (-0.00007, 0.00031) |
| Yes*12 years in practice (Reference: No*12 years in practice) | 0.0000 (-0.00014, 0.00018) |
| Yes*16 years in practice (Reference: No*16 years in practice) | -0.0001 (-0.00024, 0.00008) |
| Patient is new to PCC*Gender |  |
| Yes*Male (Reference: No*Male) | -0.0004 (-0.00160, 0.00082) |
| Yes*Female (Reference: No*Female) | -0.0074** (-0.01282, -0.00190) |
| Chronic pain diagnosis attached to encounter*Gender |  |
| Yes*Male (Reference: No*Male) | 0.0020*** (0.00110, 0.00287) |
| Yes*Female (Reference: No*Female) | 0.0261*** (0.02288, 0.02936) |
| Patient is prescribed LTOT*Gender |  |
| Yes*Male (Reference: No*Male) | 0.0036*** (0.00227, 0.00491) |
| Yes*Female (Reference: No*Female) | 0.0385*** (0.03421, 0.04286) |
| PCC previous OneSheet uses*Gender |  |
| PCC previous OneSheet uses*Male | -0.0001 (-0.00018, 0.00003) |
| PCC previous OneSheet uses *Female | -0.0004 (-0.00084, 0.00004) |

Note: Marginal effects derived from margins package in R. The margins package provides predicted marginal effects at specific values. Values for PCC years in practice represent the interquartile range. CI – Confidence interval; PCC – primary care clinician, LTOT – long-term opioid therapy; *p<0.05, **p<0.01, ***p<0.001
