# Supplementary material for: Factors associated with chronic pain clinical decision support use in primary care
Source: PLOS Digit Health. 2026 Jul 16;5(7):e0001032. doi: 10.1371/journal.pdig.0001032 (PMC13374887; doi:10.1371/journal.pdig.0001032)
Supplement: S3 Table — (DOCX) [file pdig.0001032.s003.docx]

**S3 Table.** Results of outlier sensitivity analysis

| Variable | Model 1  ME (95% CI) | Model 2  ME (95% CI) | Model 3  ME (95% CI) | Original model  ME (95% CI) |
| --- | --- | --- | --- | --- |
| *Performance expectancy* |  |  |  |  |
| Patient is new to PCC (Reference: No) | -0.00410**  (-0.00662, -0.00158) | -0.00375***  (-0.00592, -0.00158) | -0.00387***  (-0.00617, -0.00157) | 0.00419***  (0.00193, 0.00645) |
| Chronic pain diagnosis attached to encounter (Reference: No) | 0.04095***  (0.03088, 0.05102) | 0.01975***  (0.01489, 0.02460) | 0.01927***  (0.01430, 0.02424) | 0.03476***  (0.02621, 0.04331) |
| Patient is prescribed LTOT (Reference: No) | 0.05237***  (0.03974, 0.06500) | 0.05641***  (0.04328, 0.06954) | 0.0615***  (0.04699, 0.07601) | 0.04701***  (0.03565, 0.05837) |
| *Effort expectancy* |  |  |  |  |
| PCC previous OneSheet uses | 0.00036  (-0.00000, 0.00072) | 0.00008  (-0.00023, 0.00040) | 0.00014  (-0.00020, 0.00048) | 0.00030  (-0.00004, 0.00064) |
| *PCC Characteristics* |  |  |  |  |
| Years in practice | 0.00095***  (0.00081, 0.00110) | 0.00044***  (0.00030, 0.00059) | 0.00050***  (0.00034, 0.00066) | 0.00082***  (0.00045, 0.00119) |
| Male (Reference: female) | -0.00557***  (-0.00787, -0.00323) | -0.00625**  (-0.00848, -0.00402) | -0.00576***  (-0.00784, -0.00368) | -0.00414  (-0.01103, 0.00274) |
| *Testing for moderation* |  |  |  |  |
| Patient is new to PCC* Years in practice | 0.00002  (-0.00035, 0.00040) | -0.00000  (-0.00036, 0.00036) | 0.00000  (-0.00038, 0.00039) | -0.00005  (-0.00040, -0.00030) |
| Chronic pain diagnosis attached to encounter*Years in practice | -0.00044***  (-0.00057, -0.00031) | -0.00011  (-0.00023, 0.00001) | -0.00010  (-0.00023, 0.00003) | -0.00034***  (-0.00046, -0.00022) |
| Patient is prescribed LTOT*Years in practice | -0.00038***  (-0.00051, -0.00026) | -0.00039***  (-0.00052, -0.00028) | -0.00045***  (-0.00058, -0.00033) | -0.00031***  (-0.00043, -0.00019) |
| PCC previous OneSheet uses*Years in practice | -0.00003*  (-0.00005, -0.00000) | -0.00001  (-0.00003, 0.00001) | -0.00001  (-0.00003, 0.00001) | -0.00002*  (-0.00004, -0.00000) |
| Patient is new to PCC*Gender | 0.00237  (-0.00921, 0.01393) | 0.00589  (-0.00959, 0.02138) | -0.00547  (-0.00946, 0.02039) | -0.00298  (-0.00984, 0.00388) |
| Chronic pain diagnosis attached to encounter* Gender | -0.00419***  (-0.00582, -0.00256) | -0.00278**  (-0.00483, -0.00073) | -0.00249*  (-0.00479, -0.00020 | -0.00375***  (-0.00550, -0.00201) |
| Patient is prescribed LTOT* Gender | -0.00350***  (-0.00526, -0.00175) | -0.00416***  (-0.00558, 0.00274) | -0.00468***  (-0.00510, -0.00336) | -0.00281**  (-0.00475, -0.00087) |
| PCC previous OneSheet uses* Gender | -0.00007  (-0.00055, 0.00040) | 0.00005  (-0.00047, 0.00057) | -0.00005  (-0.00060, 0.00050) | -0.00004  (-0.00052, 0.00043) |
| *n* | 139,851 | 144,765 | 139,105 | 145,511 |

Note: Marginal effects derived from the mfx package in R. The mfx package provides the overall marginal effect for interaction terms. Model 1 excluded the PCC with 38 years in practice, Model 2 excluded the PCC with 42 years in practice, and Model 3 excluded both PCCs. ME – Marginal effect; CI – Confidence interval; PCC – primary care clinician, LTOT – long-term opioid therapy; *p<0.05, **p<0.01, ***p<0.001
